# The development of PROmunication: a training-tool for clinicians using patient-reported outcomes to promote patient-centred communication in clinical cancer settings

**DOI:** 10.1186/s41687-020-0174-6

**Published:** 2020-02-11

**Authors:** Pernille C. Skovlund, Sissel Ravn, Lene Seibaek, Henriette Vind Thaysen, Kirsten Lomborg, Berit Kjærside Nielsen

**Affiliations:** 1 Experimental Clinical Oncology, Department of Oncology, Aarhus University Hospital, Palle Juul-Jensens Boulevard 99, 8200 Aarhus N, Denmark; 2 The Research Centre for Patient Involvement, Aarhus University & the Central Region, Palle Juul-Jensens Boulevard 99, 8200 Aarhus N, Denmark; 3 Department of Surgery, Aarhus University Hospital, Palle Juul Jensens Boulevard, 8200 Aarhus N, Denmark; 4 Department of Gynaecology and Obstetrics, Aarhus University Hospital, Palle Juul-Jensens Boulevard 137, 8200 Aarhus N, Denmark; 5 Department of Clinical Medicine, Faculty of Health, Aarhus University, Incuba Skejby, building 2, Palle Juul-Jensens Boulevard 82, 8200 Aarhus N, Denmark; 6 DEFACTUM, Social & Health Services and Labour Market, Central Denmark Region, Olof Palmes Allé 15, 8200 Aarhus N, Denmark

**Keywords:** Patient-reported outcomes, Patient-centred communication, Training, Manual

## Abstract

**Background:**

The value of using real-time patient-reported outcome (PRO) measures in cancer communication has gained attention both in the clinic and in research. Despite this, no internationally accepted guidelines or training programs for clinicians on how to engage in patient-centred communication based on PROs exist. Lack of training may complicate implementation and systematic use of PROs in the clinic. We aimed to develop a short and feasible manual and training session in PRO-based dialogue rooted in patient-centred communication, coined PROmunication.

**Methods:**

PROmunication was implemented in two studies using PROs in different clinical cancer settings. We interviewed clinicians twice during the development phase. First, adopting a clinical perspective, they provided ideas for content, length and structure of the training session and the manual. Second, they approved the draft of the manual with minor adjustments on how to document clinician-patient communication. The final version of the PROmunication tool was built on clinicians’ input, theory on patient-centred communication, a literature review, and didactic considerations.

**Results:**

The one-page manual gave clinicians a brief and clear overview of how to prepare for, undergo and document a PRO-based consultation. Illustrations and verbal phrases were offered to operationalize and facilitate patient-centred communication. The training session included elements like evidence-based knowledge about the rationale, benefits and challenges of using PROs and comprised theory, experimental training and instructions for the use of the manual in clinical practice. Ad hoc training and feedback in the clinic followed the training session.

**Conclusions:**

This paper presents the development of a short, theory-driven manual and training session intended to support and engage clinicians in PRO-based dialogue leading to patient-centred communication. Further testing of the tool is necessary and adjustments may be required if the PROmunication tool should be applied in other clinical settings were patients are seen regularly. An evaluation of the tool is planned to be performed in future studies. Training in PROmunication may further systematic and consistent use of PRO data in the consultation, leading to patient-centred consultations and increased patient involvement.

## Background

In this paper, we introduce a new concept that combines patient-reported outcome (PRO) measures and patient-centred communication (PCC). PROs are often defined as: “any report of the status of a patient’s health condition that comes directly from the patient, without interpretation of the patient’s response by a clinician or anyone else.” [1]. PROs relate to health, quality of life or functional status associated with healthcare or treatment [2]. PCC can be defined in terms of processes and outcomes of the patient-clinician interaction: “Eliciting, understanding and validating the patient’s perspective; Understanding patients within their own psychological and social contexts; Reaching a shared understanding of the individual patient’s diagnosis and treatment options; Helping patients share decision-making power by offering meaningful involvement in choices related to their health” [3]. The new concept is coined PROmunication and indicates a switch from traditional patient-physician communication where the physician sets the agenda to a patient-centred approach based on PRO data. This switch calls for specific advice and ideas informing the implementation of PROmunication in clinical practise. To support PROmunication in outpatient cancer consultations, we developed a manual and a training session for clinicians.

### Patient-reported outcomes

The assessment of PROs is well established in quality improvement and research, particularly in clinical trials and observational studies, where they provide valuable evidence on the burden of disease and the efficacy, effectiveness, and cost effectiveness of interventions from a patient perspective [4, 5]. At an individual level, PROs can for example be used to enhance shared decision making, to focus on individual patient needs and concerns, and to monitor symptoms or disease development over time [6]. Recent digital solutions combine PRO data with an algorithm to graphically display important symptoms, functions and decision support at the individual level. The PRO data is then often visualized through colour-coded categorisation of severity such as levels of symptom severity e.g. Red: need of immediate attention; Yellow: need of response within a certain timeframe or Green: no need of response at present. In addition, patient’s needs for discussing their outcomes and preferences regarding follow-up can be captured by PROs [7].

Interest in systematic collection of PROs and awareness of the value of using real-time patient-centred data in cancer communication is growing. This awareness has led to the development of PRO tools, which have been successful in optimizing and improving patient-physician communication [8, 9]. Hence, the use of PRO data in clinical practice can improve patients’ health-related quality of life (HRQoL), initiate discussions regarding unspecific symptoms or health-related problems [10–14], improve symptom management and control [15–17]. PROs are often used as unique indicators of the impact of disease on the patient [18]. Furthermore, PROs may facilitate patient-centred consultations by providing patients with an opportunity to communicate on topics they find important and that may otherwise not be addressed [19, 20]. However, the use of PROs in the clinic is not able in itself to ensure patient involvement and patient-centred consultations [21, 22]. If clinicians do not value PROs or consider patients’ assessment of the situation and of their illness as important knowledge, then PROs are suggested not to make a difference to the dialogue [23]. When measuring the impact of PROs, several studies have been criticised for not investigating the role played by mediators, including a change in patient-physician communication and patient-centeredness as facilitators of the impact rather than PROs [24]. We anticipate that PROs used properly in the consultation has the potential to enhance patient-centred care [5].

Many studies have voiced a need for training clinicians in how to optimise, administrate, interpret and integrate PRO data in their dialogue with patients [8, 15, 19, 24–28]. If clinicians do not know how to interpret and report the PRO data or how and when to use it, they may experience PROs as a burden in clinical encounters and the use of PROs may end up doing more harm than good [28, 29]. For clinicians, the harms may include information overload, spending time on finding and reviewing PRO data and identifying problems without being able to use the data in the consultation or having access to appropriate interventions [30]. For patients, answering PRO measures without receiving any or only superficial response to the problems reported, can leave them with a feeling of being abandoned and alone, and may cause decreased motivation for filling in the PRO measures [21]. Therefore, the first step towards success is to devise implementation strategies, including training of clinicians in using PROs in a clinical context. To ensure facilitation of PCC supported by PROs, implementation strategies must be founded on PCC theory.

### Patient-centred communication

In cancer consultations, communication between clinicians and patients is a crucial part of the clinical encounter, which is characterized by the complexity of disseminating medical information and uncertainty regarding disease and treatment outcomes [31]. To understand how clinicians could support patients’ communication needs, the American National Cancer Institute commissioned an extensive literature review and report on the topic of PCC [3]. This review identifies six basic functions that cancer communication must support: Fostering healing relationships, exchanging information, responding to emotions, managing uncertainty, making decisions, and enabling patient self-management [3, 32]. These basic functions constitute competency in patient-centred communication [33] and should be the point of departure when PROs are implemented to facilitate PCC.

### Training clinicians in patient-centred communication based on patient-reported outcomes

Several models and guides have been developed for use in medical education and clinical practice to teach and assess PCC [34–40], but these models do not incorporate PROs. Furthermore, training is needed to successfully implement PROs in the consultation [8, 19, 26, 27, 41, 42], but only one study has proposed key recommendations for training clinicians to use, interpret and act on PRO data [25]. In addition, few studies have been published on developing training programs and supportive materials to teach clinicians to communicate based on PRO data [15, 19, 25, 43]. However, these are not based on PCC.

Organisations engaged in the implementation of PROs focus primarily on basic knowledge and key issues for integrating PROs in clinical practice and electronic health records and do not include the training of clinicians in communication based on PRO data [44, 45]. Recommendations from Danish PROs experts and nationwide experiences of implementing Danish PRO programs have neither been transformed into widely accepted guidelines, nor do they specify training elements regarding communication based on PROs [46, 47]. To summarize, at present there are no internationally accepted guidelines on content, structure or length of training programs for clinicians on how to communicate with patients based on PROs in a patient-centred way, nor any studies of the process of preparing for, undergoing and documenting a PRO-based consultation. Therefore, we aimed to develop a short and feasible training session and manual in PRO-based dialogue founded on PCC.

## Methods

### Setting

**Fig. 1:**
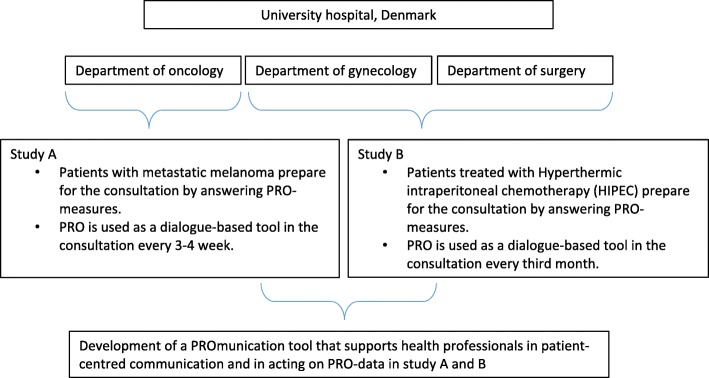
An overview of the setting in which the PRO-based communication tool was developed

The development of a PRO-based communication tool took place at a Danish university hospital as part of two research projects (study A, an intervention study and B, a feasibility study), aiming to examine the use of PROs as a communication tool in cancer consultations. The two projects were implemented at three clinical departments offering oncological and surgical treatment for metastatic cancer (Fig. 1).

In study A, which began 6 months prior to study B, consultations were performed every 3–4 weeks during treatment, whereas in study B, patients attended quarterly consultations after their surgery. Before any planned consultation, all patients prepared themselves by answering questions addressing clinically relevant PROs. The answers were submitted directly into their electronic health record and visualized for clinicians in green, yellow, orange or red according to level of severity. Level of severity was based on PRO scales where each item had a scale consisting of four steps ranging from “Not at All - A Little - Quite a Bit - Very Much”. This real-time PRO feedback enabled clinicians to prepare for a dialogue with patients on issues important to them. None of the physicians at the departments involved used PROs in consultations prior to the implementation of the PRO-based communication tool.

To indicate that communication based on PROs in cancer consultations was different from ordinary communication with cancer patients, the new concept “PROmunication” was coined. To convey the proper way of communication based on PROs, an appertaining PROmunication tool was developed before the intervention (study A and B).

### Design

The PROmunication tool was based on four elements: 1) a theoretical take-off grounded in a framework for patient-clinician communication, 2) empirical knowledge, 3) clinician involvement and 4) didactic considerations. In the following, we only state patient even though PROmunication includes communication with both patients and their relatives where this is relevant.

#### Theoretical take-off

As a first step towards a theory-driven tool to support PCC based on PROs, the underlying concept was clarified by defining a theoretical framework for PCC [3]. This framework includes six somewhat overlapping and interacting core functions, all serving as underlying components in the PROmunication tool:

- **Fostering healing relationships** where patient and clinician acknowledge each other’s role in mutual trust, openness and respect
- **Exchanging information** in the reciprocal efforts of both clinician and patient to obtain a shared understanding of the medical and personal issues underlying the patient’s health condition
- **Responding to emotions** by using verbal expressions of understanding, legitimation, empathy and support to address patients’ emotional distress appropriately and directly
- **Managing uncertainty** by framing information in terms of what is known and what is unknown and acknowledging the unavoidable uncertainty
- **Making decisions** preferably based on the patient’s values and understanding of the evidence and rationale for the decision conveyed by the clinician and made in agreement
- **Enabling patient self-management** by facilitating patients’ capacity to solve health-related problems and to take actions to improve their health

These six core functions offer the possibility to operationalise the construct of PCC [48], however several limitations to comprehensively assess PCC exist. First, many questionnaires measuring communication are not grounded in a conceptual model of PCC and address only specific elements of the interaction process (e.g. shared decision-making or patient autonomy support). Second, the assessment method vary greatly among studies, and third, the results are inconsistent across measurements [48]. The theoretical framework chosen for the present study could be criticised, because it was founded on studies that may not have assessed PCC comprehensively. However, the core functions are believed to constitute “best practice” for physician communication in medical encounters [33]. Since the theoretical framework for PCC was developed in cancer care, it matched our setting and was considered an appropriate theoretical background.

#### Empirical knowledge

A literature search was performed in order to gain empirical knowledge on models of and considerations on the training of clinicians to integrate PROs in clinical practice. We searched PubMed, CINAHL and PsycINFO and subject headings searches were conducted combining terms for clinician (e.g. physician, practitioner), training (e.g. education, teaching, guiding) PROs (e.g., patient-reported outcome, self-reported questionnaire), and clinical practice (e.g. consultation, routine practice). To our knowledge, training clinicians to use PROs in clinical practice rest partly on experience communicated in four studies. One study investigated when and how to use and interpret PROs in child mental health [43]. This study showed that clinicians attending a three-day training course were more positive towards PROs and had higher levels of self-efficacy regarding administering PROs and using feedback from PROs than clinicians attending a one-day course or no training.

Based on earlier experiences with the use of PROs to allow patients to elaborate on their self-perceived problems, another study highlighted training that included strategies on how to incorporate an explicit reference to PROs in the standard medical interview, and management guidelines for symptoms that were considered difficult to manage (e.g. fatigue) [15].

In a third study, operational recommendations were developed grounded on experiences from three different training programs for clinicians prior to the implementation of PROs. The recommendations include: 1. Engaging clinicians, 2. Brief training to fit practices, 3. Experimental problem-based training, 4. Decision-support aids, 5. Feedback, and 6. Engaging multidisciplinary teams [25]. These recommendations are generic and therefore serve as a guiding model, but the content of the guidelines still need to be developed according to local practice.

A fourth study describes the creation of a PRO implementation program serving as a standardised approach for the use of PROs in a clinical setting. Here, training was believed to be essential to successful PRO implementation and should contain: 1. Demonstrations on how PROs fits into a clinical work flow, 2. Instructions on where the PRO results are located within the electronic health record, 3. Education about which scores indicate the need to intervene, and 4. Training to acknowledge the completion of PROs [19].

Findings from these four studies and theory on PCC were used to establish a template of the content and length of our training and manual. Based on the literature, we aimed at engaging clinicians in developing a one or two-day training course with experimental problem-based training (examples of videotaped cases of clinician-patient interactions based on PRO) and followed up by feedback. The training should include clinical management guidelines, instructions on where to locate the PRO results within the electronic health record and a clarification of the importance of explicit reference to PRO. Participants should be both physicians and nurses.

#### Clinician involvement

To optimise implementation, ease-of-use, and to follow the literature guidelines, promote acceptance and commitment, clinicians were involved in the development process. Three physicians, one male and two females aged from 46 to 55, with different rank and experience participated in the development of the PROmunication tool. The clinicians all had several years of expertise from oncology and were recruited because they were expected to be the three main users of the tool in research project, study A. The clinicians were interviewed twice during the development phase. Notes were taken from both interviews and were used to reform the ideas within the author group. The first interview was based on a template of the manual conducted by all authors and on a template of the training session template conducted primarily by PCS and BKN but with inspiration from SR, LS and HVT. Thus, both the manual and the training session reflected a elaborated their clinical perspective for content, length, and structure of both training session and manual. To ensure that use of the PROmunication tool would be compatible with organisational requirements, the clinicians argued that the training session should be no longer than 1 h and cover the following topics: information on either study A or B, definition of PROs, how to obtain access to the PRO data, how and when to refer to PROs in the consultation, and finally how to document the use of PRO data. They did not opt for clinical management guidelines because they preferred flexibility in assessing the individual patient’s specific needs. In addition, they argued for an informal feedback right after the manual had been used. For the clinicians, the overall purpose of the manual was to provide a brief overview of the core elements within the training session in a format easy to apply and intuitive to use. For that purpose, the manual was suggested to be no longer than one A4 page. The clinicians were not asked and did not comment on didactic aspects, e.g. the composition of participants or who should perform the training. At the second interview, a tool prototype was presented to the clinicians, which led to a discussion on how to approve and document the use of PROs within the electronic health record. The clinicians approved the prototype with minor adjustments in relation to documentation of the dialogue. The manual was found to accommodate relevant needs, be easy to read and beneficial in communication with patients.

#### Didactic considerations

To cover the topics raised by the clinicians, we chose author BKN (psychologist) and HVT (clinical nurse specialist), who were both knowledgeable about study A or B, PROs, and PCC to perform the training.

The learning goals for participants to achieve were:

A. To be familiar with core elements of PROs and PROmunication, including but not limited to how to prepare, conduct and document PROmunication
B. To gain knowledge and be enthusiastic about the use of the manual

The training session and the manual were provided before implementing PRO in clinical practice and will be described in detail below.

## Results

The following section describes the development of PROmunication. Overall, the authors composed a template that were refined by the comments and ideas from the interviews with the clinicians. Especially their proposed length was different from our original template, but that also induced limitations for the consent. We chose to exclude examples of videotaped cases and had to rethink the problem-based training in order to keep it simple for the clinicians.

### Manual

**Fig. 2:**
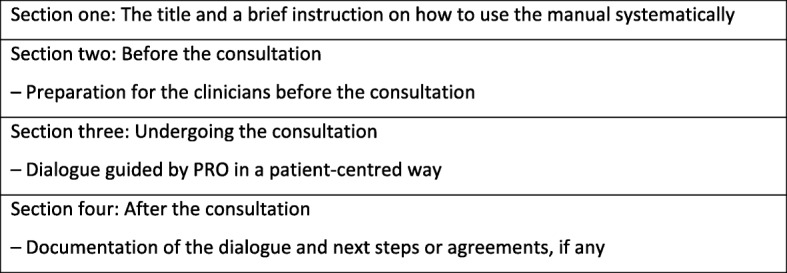
The main content of each section in the manual

The main purpose of the manual was to give a brief and clear overview of the core elements of the training session and, especially, how to prepare for, undertake, and document the consultation based on PROs. To ease use and ensure that consultations were performed systematically and focused on patient needs and preferences, the manual had a brief instruction to PROs in the consultation and three action-oriented sections with direct instructions on preparing for, undertaking and documenting the consultation. Figure 2 illustrates the main contents of each section. The final version of the manual can be found in Danish and English (see Additional file 1 and Additional file 2).

**Fig. 3:**
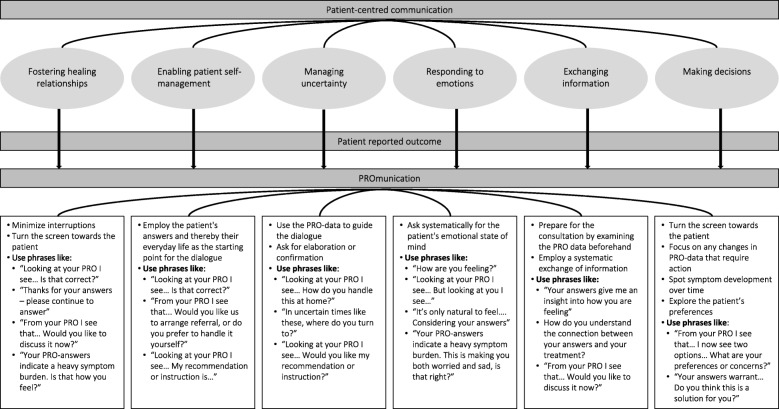
PROmunication as a means of patient-centred communication through patient-reported outcomes

To help the clinician find the PRO data and prepare prior to the patient consultation, three illustrations are shown in the section concerning preparation for the consultation; one illustrates a selection of questions and patient-answers on a Likert scale; another one illustrates how to locate PROs in the electronic health record;, and the last one gives an example of how PROs are displayed in the electronic health record. In the section about undertaking the consultation, specific examples on verbal phrases that will lead to elaboration, clarification and use of PROs to guide the dialogue are displayed in speech bubbles. Thus, the six core functions of PCC are operationalised into phrases or actions that could be used in clinical practice when PROs were fed back to clinicians. Figure 3 offers examples of phrases ready for a clinical dialogue based on PROs and reflecting PCC. To save space, not all phrases in Fig. 3 are contained in the manual.

PROmunication acts as a means of achieving patient-centred communication through patient-reported outcomes. For instance, by asking the patient: “Looking at your PROs, I see … Is that correct?” the clinician indicates that the PROs have been read, but the patient is offered a chance to elaborate and clarify. This approach may foster patients’ self-management by helping them reflect and verbalise their own condition and by facilitating a discussion about potential solutions. Further, self-management is fostered by supporting patients’ autonomy by appreciation of the patient’s perspective and attribution of the patient’s capability. The approach may also contribute to fostering healing relationships by indicating interest and trust. In addition, a consultation based on thoughts and concerns of the patient may leave the patient feeling acknowledged and respected. Additionally, turning the screen towards the patient indicates that the patients’ answers are important and that an active engagement by patients is endorsed. This should invite for shared decision-making. The last section about documentation is short and operational. Only if something new is decided or planed based directly on PROs should this be documented in the electronic health record for other clinicians to find.

The final manual was a short, one-page document as requested by the clinicians. Action-oriented sections were highlighted by illustrations and the same colour range as in the electronic health record and the PRO platform with arrows showing the direction from before to during and after the consultation indicating the process during the consultation. The layout of the manual and the verbal examples facilitated clinical practice implementation. At the bottom of the manual, we added contact information to the team behind the PROmunication tool.

### Training session

The training session covered the following elements:

1. Evidence-based knowledge about the rationale of using PROs in clinical healthcare services
2. Information about the benefits of using PRO data in the consultation.
  - PRO data enable the patient and the clinician to prepare for the consultation
  - The dialogue can focus on what is most important for the patient to discuss
  - PRO data may foster awareness of issues that are considered difficult to manage, yet importance to discuss
3. Knowledge about study A or B, for which the PROmunication tool was developed
4. Experimental training: answering the same outcome measures that the patients are to complete before the consultations. This was done to become familiar with the questions in the PROs
5. Knowledge about how to inform patients about PRO with regard to voluntary participation, accessing PRO data, time frames and assumed benefits
6. Instruction on how to use the PROmunication manual
  - Where to find the PRO data in the electronic medical chart (interface)
  - How to prepare for the dialogue by going through the PRO data
  - How to refer to PROs by explicitly mentioning and expressing one’s gratitude to their reply (e.g. “Thank you for your reply. The answers give me an idea of how you are doing”) and some specific sentences that could open up the dialogue (e.g. “Let us now talk about your replies”), reassure or clarify the meaning of the replies or invite the patient to elaborate (e.g. “It looks as if … Is that correct?”). The operationalisation of the six core functions of PCC shown in Fig. 3 served as examples of how to refer to PROs in a patient-centred way.
  - How to document the use of PRO data within the consultation

The training session was supported by a 40-min slide presentation including the experimental training and 20 min for questioning and discussions. Special attention was given to explaining the manual as this would be the future reference for the clinicians. To make the presentation vivid and easy to remember, photos and illustrations had the same layout as the manual. After implementation of the PROmunication tool, a representative of the development team was responsible for follow-up on training, including ad hoc training, feedback and discussions.

## Discussion

As demonstrated above, the theory-driven PROmunication tool consisting of a manual and a training session was developed to guide the PRO-based dialogue toward PCC. We have operationalized the six core functions of PCC into phrases and actions suitable in the context of digital solutions where PROs are supposed to promote PCC. Instructions on turning the screen towards the patient, employing the patients’ answers and everyday life as a starting point and guidance for the dialogue, and asking for elaboration are all elements that contribute to a patient-centred approach. The training session covers these instructions and communication ideas and the manual clearly features some of them to apply in in clinical context. We therefore advance that using the manual after the training session can constitute a platform to perform PCC. Several PCC models have been developed, and several studies have highlighted the importance of training clinicians in the use and interpretation of PROs [15, 19, 25, 43], but this is the first study to attempt an integration of both PROs and PCC in the training.

PROmunication offers a generic approach to promoting PCC in consultations. The generic nature of the tool was a deliberate choice because what characterise cancer care (the complexity of disseminating medical information and uncertainty regarding disease and treatment outcomes) could also characterise other diagnoses. Obviously, PROs must be adjusted to the actual disease or patient group, but the principles of engaging in communication based on PRO data are the same. Additionally, the fact that the PROmunication tool was developed for consultations between clinicians and patients from both medical and surgical settings suggests that it may also be adapted for non-cancer settings were patients are seen regularly as part of medical check-ups or follow-up programmes. Healthcare professions other than physicians may also benefit from PROmunication. Nurses, physiotherapists and psychologists also have unassisted consultations where PROs could be relevant. Researchers from different disciplines (medicine, psychology and nursing) participated in the process of developing the PROmunication tool and contributed with their specific professional knowledge to the final tool. This may ease transfer of the tool to other healthcare professions. Still, further testing is necessary to determine the scope of PROmunication.

It is anticipated that the short training session combined with the manual contributes to an easy and systematic use of PROs for all clinicians and fit a clinical workflow where new implementations need to be converted into practice quickly to further sustainability in consultations. The generic nature of the PROmunication tool necessitates adoption of a strategy allowing a constant flow of medical students and other healthcare professionals to be trained without spending too much time or money on training. It can be discussed whether longer and more intense training sessions are more efficient than short training and ad hoc follow-up. A Cochrane review on interventions for healthcare professionals to promote a patient-centred approach in clinical consultations concluded that short-term training (less than 10 h) was as successful as longer training [49]. Longer training could also limit participation to only the most motivated clinicians [50]. The one-page manual made an easy brush-up possible for trained clinicians, and new clinicians could easily get a quick understanding of the principles. Additionally, it can be discussed in which way cancer patients may benefit from clinicians being taught communication skills. Despite clinicians’ training in for instance increasing open questions or posing empathic statements, reviews did not find convincing evidence for impacting neither patient satisfaction nor distress [49, 51]. However, other studies suggest a positive effect on patient satisfaction when initiatives target both doctors and patients [52] or when clinicians use positive communication behaviors (e.g. begin with open-ended question) [53].

Use of targeted PCC strategies such as PROmunication do not necessarily make all patients feel at ease and encouraged to ask medical questions. In fact, a study on patients with hypertension showed that patients with low health literacy levels asked the physician fewer medical questions than patients with higher levels of health literacy even if the clinicians had received PCC skills training [54]. For PCC to unfold on the basis of PROmunication, the selected PROs must capture the patient’s perspective [55] and be relevant, meaningful and adequate for all involved parties, namely the clinician, the patient and patients’ relatives [56]. Clinician and patient preferences should be balanced in selecting the questionnaire and type of PRO data to collect [5, 7, 57]. If PROs are inappropriate for a diagnosis or patient group, then the PCC will likely have limited use. Language, cultural appropriateness, the time and burden put on patients and patients’ health literacy level should also be considered when PROs are chosen [58].

The development process and the final PROmunication tool should be considered in the context of its strengths and limitations. It is a strength that using PROmunication establishes alignment for clinicians of what exactly is expected before, during, and after the consultation for PROs to be patient-centred. Another strength is that when used systematically, PROmunication implies mutual agenda-setting in the consultation at the patient’s terms, thereby facilitating activated and involved patients, and a focus on the patient as a whole person not just a disease. Limitations include absence of patient involvement in the development process. Patients could have contributed with their perspectives on the PROmunication tool and potentially also validated or dismissed some of our examples on verbal phrases that would lead to elaboration, clarification and use of PROs to guide the dialogue. In addition, lack of a pilot study or an evaluation plan before the implementation of PROmunication, limits the knowledge of the effectiveness of PROmunication. Neither the feasibility nor the capability of the tool to promote PCC has been systematically tested, but an evaluation will be made in the two research projects (study A and study B). In these two studies, patients’ self-assessed level of involvement in the consultation (e.g. involvement in decisions, sincere interest in the patient shown by the clinicians and a focus on what the patient finds important) will be determined as an indicator of patient-centeredness and PCC. Another way to evaluate the PROmunication tool could be to ask the clinicians to what extent the learning objectivities were met and used in practice. If an evaluation suggests a refinement of the PROmunication tool, patients’ perspective on the tool could be captured in the refinement process. A review from 2018 shows that using PROs in the care for individual patients supports patients in raising issues with clinicians, but this does not substantially change clinicians’ communication practices with patients [8]. Another review from 2013 found that when clinicians undergo communication skills training, they are more likely to use open questions and show empathy towards patients than clinicians who have not received training. However, the review did not examine to which extent this effect was sustained, nor did it determine the need for consolidation sessions and which types of communication skills training were most likely to work [59]. Additionally, the durability of the training in PROmunication also needs to be tested, and longitudinal studies that follow clinicians over time may be beneficial in assessing the effectiveness and durability of communication skills training [60]. Another limitation is that PCC may be more important to some patients than others [61], but this has not been studied in relation to PROmunication. The training session within the PROmunication is limited to clinicians. Patients received no formal training in how to engage in consultations. Paired communication training for both patients with advanced cancer and oncologists may improve patient-centred communication [50] and there is some indication that interventions including condition-specific educational materials that aim to promote patient-centred care within clinical consultations have beneficial effects on health behaviour and health status [49]. Finally, even though the clinician-patient-relative relationship and nonverbal interaction between the parties are important facilitators to PCC [62], the PROmunication tool did not focus on these issues. Section two of the manual contains a phrase about avoiding interruptions, hence indirectly acknowledging the importance of the setting and verbal and nonverbal behaviour to achieving PCC [3].

## Conclusion

This study presents the development of a short, theory-driven manual and training session termed PROmunication, aiming to support and engage clinicians in PRO-based dialogue with patients and to align, systematize and patient-centre the way clinicians communicate in cancer consultations. PROmunication was applied in two different clinical cancer settings; and with slight adjustments, it may facilitate PCC in other clinical settings as well. It is expected that training in PROmunication will further systematic use of PRO-data in the consultation leading to patient-centred consultations and thereby fostering healing relationships.

## Future perspectives

The PROmunication tool should be tested in other patients than cancer patients, in other care processes and in other professions than physicians. Particularly interesting would be to study the use of the PROmunication tool over time to appraise its sustainability or to study whether PCC leads to a beneficial use of PRO. Further attention should also be paid to studies of the costs and time required to adjust the PROmunication tool to other settings, to train clinicians and to maintain and update the PROmunication tool. PROmunication could be relevant and applied in an educational context to illustrate how PCC may be facilitated through PROs.

## Supplementary information

**Additional file 1.** The original manual.

**Additional file 2.** The manual translated into English.

## Data Availability

Data sharing is not applicable to this article as no datasets were generated or analyzed during the current study.

## Abbreviations

PCC: Patient-centered communication
PROs: Patient-reported outcomes
