# Supplementary material for: The development of PROmunication: a training-tool for clinicians using patient-reported outcomes to promote patient-centred communication in clinical cancer settings
Source: J Patient Rep Outcomes. 2020 Feb 11;4:10. doi: 10.1186/s41687-020-0174-6 (PMC7013008; doi:10.1186/s41687-020-0174-6)
Supplement: Supplementary file 1 — Additional file 1. The original manual. [file 41687_2020_174_MOESM1_ESM.pptx]

## Slide 1
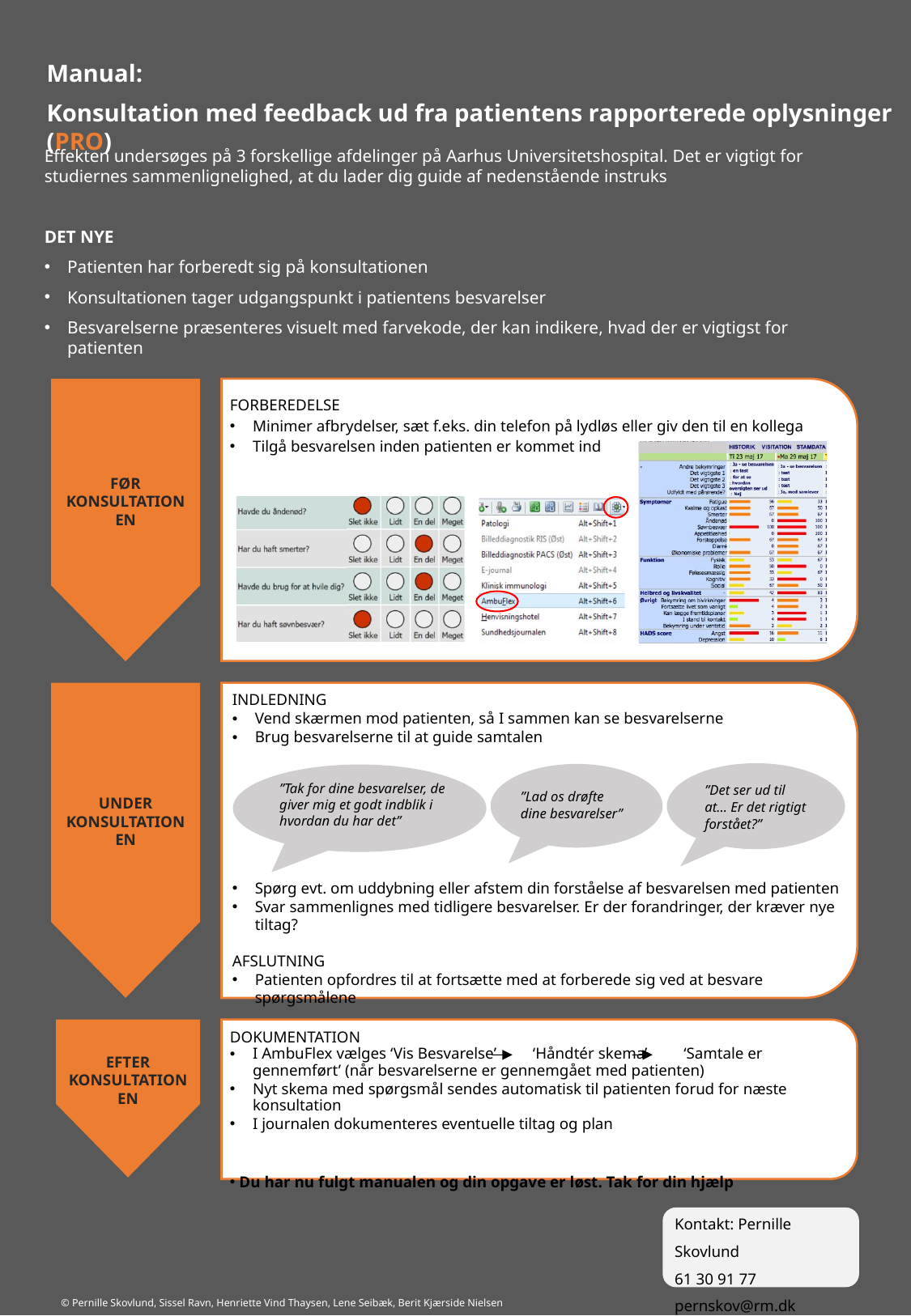

Manual:
Konsultation med feedback ud fra patientens rapporterede oplysninger (PRO)
Effekten undersøges på 3 forskellige afdelinger på Aarhus Universitetshospital. Det er vigtigt for studiernes sammenlignelighed, at du lader dig guide af nedenstående instruks
DET NYE
Patienten har forberedt sig på konsultationen
Konsultationen tager udgangspunkt i patientens besvarelser
Besvarelserne præsenteres visuelt med farvekode, der kan indikere, hvad der er vigtigst for patienten
FORBEREDELSE
Minimer afbrydelser, sæt f.eks. din telefon på lydløs eller giv den til en kollega
Tilgå besvarelsen inden patienten er kommet ind
FØR KONSULTATIONEN
INDLEDNING
Vend skærmen mod patienten, så I sammen kan se besvarelserne
Brug besvarelserne til at guide samtalen
Spørg evt. om uddybning eller afstem din forståelse af besvarelsen med patienten
Svar sammenlignes med tidligere besvarelser. Er der forandringer, der kræver nye tiltag?
AFSLUTNING
Patienten opfordres til at fortsætte med at forberede sig ved at besvare spørgsmålene
”Det ser ud til at… Er det rigtigt forstået?”
”Lad os drøfte dine besvarelser”
UNDER KONSULTATIONEN
”Tak for dine besvarelser, de giver mig et godt indblik i hvordan du har det”
DOKUMENTATION
I AmbuFlex vælges ‘Vis Besvarelse’ ‘Håndtér skema’ ‘Samtale er gennemført’ (når besvarelserne er gennemgået med patienten)
Nyt skema med spørgsmål sendes automatisk til patienten forud for næste konsultation
I journalen dokumenteres eventuelle tiltag og plan
 Du har nu fulgt manualen og din opgave er løst. Tak for din hjælp
EFTER KONSULTATIONEN
Kontakt: Pernille Skovlund
61 30 91 77 pernskov@rm.dk
© Pernille Skovlund, Sissel Ravn, Henriette Vind Thaysen, Lene Seibæk, Berit Kjærside Nielsen
