# Supplementary material for: The development of PROmunication: a training-tool for clinicians using patient-reported outcomes to promote patient-centred communication in clinical cancer settings
Source: J Patient Rep Outcomes. 2020 Feb 11;4:10. doi: 10.1186/s41687-020-0174-6 (PMC7013008; doi:10.1186/s41687-020-0174-6)
Supplement: Supplementary file 2 — Additional file 2. The manual translated into English. [file 41687_2020_174_MOESM2_ESM.pptx]

## Slide 1
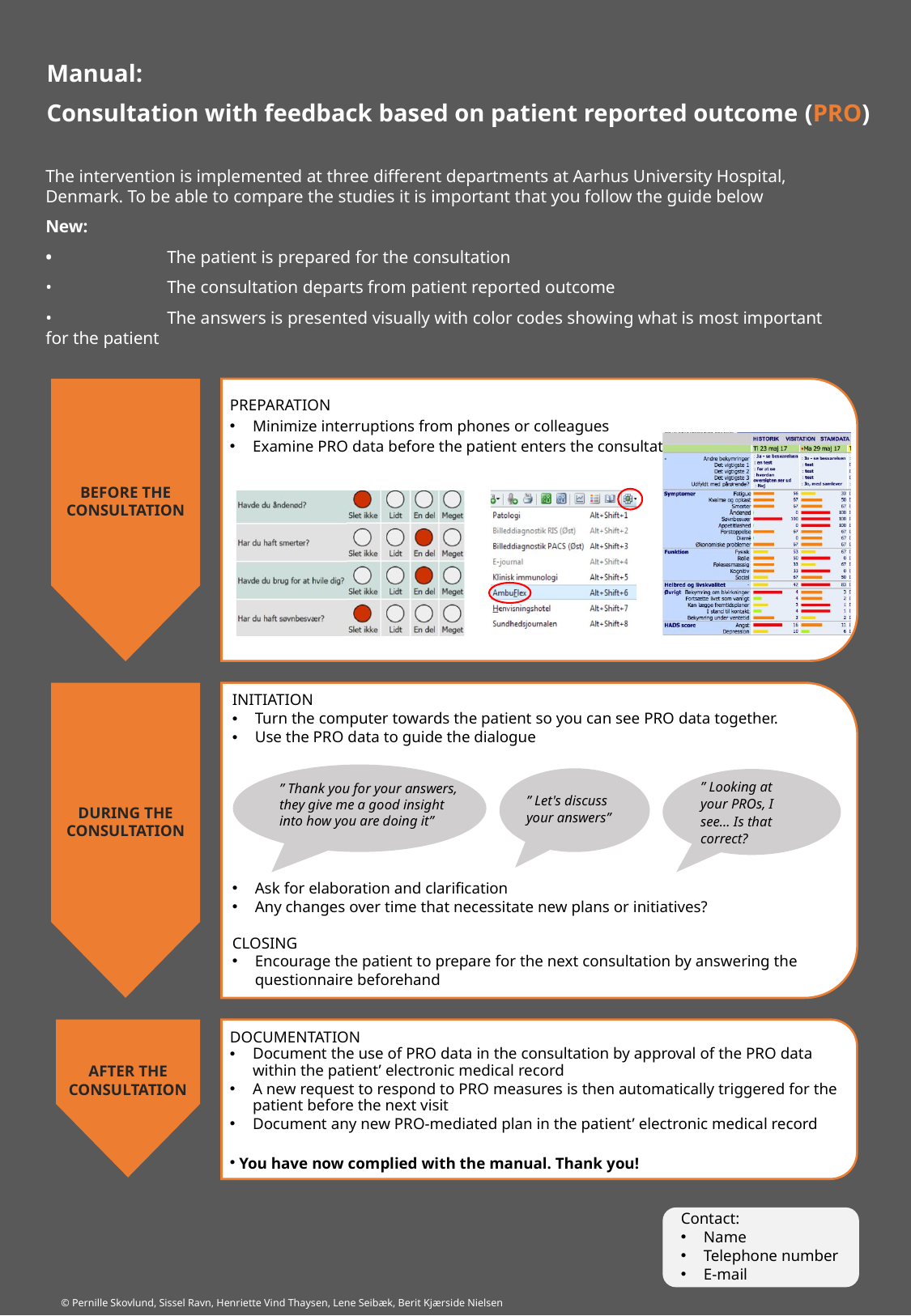

Manual:
Consultation with feedback based on patient reported outcome (PRO)
The intervention is implemented at three different departments at Aarhus University Hospital, Denmark. To be able to compare the studies it is important that you follow the guide below
New:
•	The patient is prepared for the consultation
•	The consultation departs from patient reported outcome
•	The answers is presented visually with color codes showing what is most important for the patient
PREPARATION
Minimize interruptions from phones or colleagues
Examine PRO data before the patient enters the consultation
BEFORE THE CONSULTATION
INITIATION
Turn the computer towards the patient so you can see PRO data together.
Use the PRO data to guide the dialogue
Ask for elaboration and clarification
Any changes over time that necessitate new plans or initiatives?
CLOSING
Encourage the patient to prepare for the next consultation by answering the questionnaire beforehand
DURING THE CONSULTATION
” Let's discuss your answers”
” Looking at your PROs, I see… Is that correct?
” Thank you for your answers, they give me a good insight into how you are doing it”
DOCUMENTATION
Document the use of PRO data in the consultation by approval of the PRO data within the patient’ electronic medical record
A new request to respond to PRO measures is then automatically triggered for the patient before the next visit
Document any new PRO-mediated plan in the patient’ electronic medical record
 You have now complied with the manual. Thank you!
AFTER THE CONSULTATION
Contact:
Name
Telephone number
E-mail
© Pernille Skovlund, Sissel Ravn, Henriette Vind Thaysen, Lene Seibæk, Berit Kjærside Nielsen
